# Ocular Biodistribution of Once-Daily 0.6% Bilastine Eye Drops Reveals Highest Levels in Conjunctiva Up to 24 h Postadministration

**DOI:** 10.1089/jop.2022.0024

**Published:** 2022-11-08

**Authors:** Inés Torrens, Álvaro Ganza, Gonzalo Hernández, Ana Gonzalo, Arturo Zazpe

**Affiliations:** Research, Development and Innovation Department (R&D+i Department), Faes Farma, Bizkaia, Spain.

**Keywords:** bilastine, antihistamines, allergic conjunctivitis, pharmacokinetics, biodistribution, LC-MS/MS

## Abstract

**Purpose:**

Bilastine is a second-generation antihistamine that has been shown to be effective for treatment of allergic conjunctivitis. The objective of this study was to evaluate the pharmacokinetics (PKs) and biodistribution of 0.6% bilastine preservative-free eye drops.

**Methods:**

Bilastine was quantified in the conjunctiva, cornea, aqueous humor, vitreous humor, iris/ciliary body, retina/choroid, crystalline lens, and plasma, following a single topical administration to male Dutch-belted rabbits.

**Results:**

Concentrations of bilastine were highest in the conjunctiva [C_max_: 2,545.04 ng/g, at 6 h postadministration; area under the concentration–time curve (AUC_t_): 11,382.40 ng·h/g] and cornea (C_max_: 609.11 ng/g, at 1 h postadministration; AUC_t_: 1,993.88 ng·h/g), followed by the iris/ciliary body, retina/choroid, aqueous humor, plasma, vitreous humor, and crystalline lens. Quantifiable bilastine concentrations were observed up to 24 h after instillation in the conjunctiva (388.45 ng/g), cornea (28.68 ng/g), iris/ciliary body (12.42 ng/g), retina/choroid (1.91 ng/g), and crystalline lens (0.12 ng/g). In plasma, aqueous humor, and vitreous humor, bilastine was detected up to 12 h postadministration (0.18 ng/mL, 0.40 ng/mL, and 0.32 ng/g, respectively).

**Conclusions:**

PKs and biodistribution of 0.6% bilastine eye drops in rabbits revealed a marked preferential distribution in the conjunctiva (target tissue), with sustained levels up to 24 h. These findings are consistent with clinical efficacy trials supporting once-daily administration of topical bilastine for treatment of allergic conjunctivitis.

## Introduction

Ocular allergy is a common inflammatory condition that has dramatically increased in the last decades and continues to rise.^1,2^ The hallmark signs and symptoms have been estimated to be present in 40%–80% of the allergic population,^3,4^ including itching, redness, tearing, swelling, and vision loss in severe cases such as atopic keratoconjunctivitis.^5,6^

Despite the increase in prevalence of allergic diseases, allergies of the eye are often underdiagnosed and subsequently undertreated.^7^ The most frequent clinical forms of ocular allergy are seasonal allergic conjunctivitis and perennial allergic conjunctivitis, which are usually mild.^8^ The primary goal in their management is to identify the potential allergens causing the symptoms so that they can be more easily avoided.^8^ However, this is not easy to achieve, and medication is used early to minimize symptoms of the allergic reaction and to avoid chronicity of the disease.

Current drug therapies are mainly focused on the key mechanisms involved in development of the disease. For mild to moderate conditions, an initial therapy may include topical antihistamines (including cetirizine and levocabastine, among others), mast cell stabilizers, and dual-acting agents (topical mast cell stabilizers and antihistamines, such as olopatadine, azelastine, and ketotifen), whereas chronic and severe allergic eye disorders are a challenge for treatment.^9,10^

In general, topical ocular drugs are the first choice of treatment for allergic conjunctivitis given their fast onset of action and direct targeting of the eye, resulting in higher bioavailability than systemically administered drugs.^10^ In addition, topical eye drops are considered the most convenient, safe, patient-compliant, and noninvasive route of ocular drug administration.^11^

Given the role of histamine in allergic responses, the use of H1-antihistamines is recommended for patients with allergic conjunctivitis. H1-antihistamines can act either as neutral receptor antagonists or inverse agonists. Receptor antagonists bind to the receptor and block the activation of the receptor by histamine, whereas some H1-antihistamines also act as inverse agonists, binding and stabilizing the inactive form of the receptor, and downregulate constitutive receptor activity.^12,13^

Therefore, H1-antihistamines with inverse agonist activity are more potent than neutral antagonists as they suppress this intrinsic signal in addition to the H1-antihistamine effect. Second-generation antihistamines have been developed to reduce or eliminate the sedation and anticholinergic adverse effects associated with first-generation H1-receptor antagonists.^10^

Bilastine is a nonsedating second-generation H_1_-antihistamine characterized by a fast onset of action and a prolonged effect,^14,15^ with inverse agonist activity.^16^ To date, bilastine has been administered through the oral route in the symptomatic treatment of seasonal or perennial allergic rhinoconjunctivitis and urticaria.^17^ Recently, a new ophthalmic formulation of bilastine has been developed for the treatment of allergic conjunctivitis. Its safety, good tolerability, and effective once-daily dosing have been demonstrated in two clinical trials (clinical trials NCT03231969 and NCT03479307).^18^

The aim of the present preclinical study was to perform pharmacokinetic (PK) and biodistribution studies to determine the time course of bilastine concentration in plasma and ocular tissues (conjunctiva, cornea, aqueous humor, iris/ciliary body, crystalline lens, vitreous humor, and retina/choroid) by liquid chromatography–tandem mass spectrometry (LC-MS/MS) after a single ocular administration of 0.6% bilastine ophthalmic solution to male Dutch-belted rabbits.

## Methods

All experimental and animal handling procedures were performed in accordance with the Association for Research in Vision and Ophthalmology (ARVO) Statement on the Use of Animals in Ophthalmic and Vision Research, in agreement with OECD Principles of Good Laboratory Practice ENV/MC/CHEM(98)17. The study was approved by the Ethics Committee on Animal Experimentation of Envigo CRS.

### Animals

A total of 24 male Dutch-Belted rabbits were used in the study. At treatment, the animals were ∼4–5 months of age and their body weights ranged from 1.6 to 2.1 kg. At arrival and before treatment, ophthalmic examinations of the animals were performed by a veterinary surgeon. Only animals with no ocular abnormalities were included in the study. The rabbits were acclimated to study conditions for 13 days before dose administration.

Animals were housed individually, with food and water *ad libitum*, in rooms under controlled temperature (19°C–21°C) and humidity (60%–80%), which had ventilation rates of 14 air changes per hour. Rabbits were kept under standard 12-h light–12-h dark cycles throughout the study. Animals were weighed before administration and observed after administration to record any possible clinical sign.

### Dose administration and blood sampling

Each animal received 30 μL of bilastine solution at 6 mg/mL on a single occasion in both eyes (total bilateral dose of 60 μL). The dose was administered with an automatic pipette and new pipette tips were used for each eye. Following dose administration, blood samples (0.5 mL) were obtained through the ear artery at prespecified time points: 0.5, 1, 2, 4, 6, 8, 12, and 24 h.

Blood samples were collected into lithium heparin anticoagulant polypropylene tubes and subsequently centrifuged at 2,300 *g* for 10 min at 4°C. Resulting plasma was divided into two aliquots of at least 100 μL each, immediately frozen in dry ice, and stored at −80°C ± 10°C until analysis.

### Specimen collection

After blood sample collection, three animals were sacrificed at each time point by intravenous injection of sodium pentobarbital into the ear vein. Immediately after euthanasia, the following samples from both eyes (right and left eyes, processed independently) were collected: aqueous humor, vitreous humor, cornea, conjunctiva, iris/ciliary body, crystalline lens, and retina/choroid. The solid matrices were collected into previously tared containers and then carefully weighed to determine the amount of each tissue collected.

Once weighed, each solid matrix was transferred to a DT-20 tube (IKA^®^, Staufen, Germany) containing 5 mL of acetonitrile (ACN) for the retina/choroid; 5 mL of methanol (MeOH) for the conjunctiva, cornea, and iris/ciliary body; and 5 mL of MeOH/ACN (1:1, v/v) for the crystalline lens. Tubes were then placed in dry ice and stored at −20°C ± 5°C until analysis. Aqueous humor and vitreous humor were aliquoted in cryotubes, immediately placed in dry ice, and stored at −80°C ± 10°C until analysis.

### Sample preparation and extraction method

On the day of the analysis, eye matrices and plasma samples were thawed at room temperature. Plasma samples (75 μL) were placed in 1.5-mL Eppendorf tubes and 225 μL of internal standard (IS, bilastine d6) solution at 0.01 μg/mL was added. The mixture was vortexed for 30 s and centrifuged at 14,000 rpm for 5 min at 4°C. The supernatant was transferred to a 96-well plate, centrifuged at 3,500 rpm for 5 min at 4°C, and injected into the LC-MS/MS system.

Aqueous and vitreous humor samples (20 μL) were transferred to Eppendorf tubes, and 100 μL of IS solution at 0.005 mg/mL was added. Due to its high viscosity, vitreous humor samples were deposited into previously tared cryotubes and weighted. The mixtures were vortexed for 30 s and centrifuged at 14,000 rpm for 5 min at 4°C. Supernatants were collected and injected into the LC-MS/MS system. Cornea, iris/ciliary body, conjunctiva, crystalline lens, and retina/choroid samples were homogenized using an Ultra-Turrax homogenizer at maximum speed for 10 min.

Homogenates (100 μL) were transferred to a polypropylene tube and centrifuged at 3,500 rpm for 10 min at 4°C. Then, 50 μL of the supernatants was transferred to an Eppendorf tube. Subsequently, 20 μL of IS solution at 0.006 μg/mL and 5 μL of MeOH were added and vortexed for 30 s and centrifuged at 3,500 rpm for 5 min at 4°C. Supernatants were collected and injected into the LC-MS/MS system for analysis.

### LC-MS/MS analyses

Bilastine concentrations were determined using LC-MS/MS methods for all eye matrices using a stable isotope label (bilastine-d6) as the IS. Analyses were performed at Eurofins ADME BIOANALYSES (Vergèze, France) on a Shimadzu high performance liquid chromatography (HPLC) system (Shimadzu, Columbia, MD, USA) coupled to an AB Sciex API 5500 Qtrap mass spectrometer (AB Sciex, Concord, ON, Canada), equipped with an electrospray ionization source. HPLC analysis was performed on a Kromasil 100–5C18 column (50 × 3.0 mm) eluted with different gradients (depending on the matrix analyzed) of ammonium acetate/water/ACN at a flow rate of 0.8 mL/min.

### Analytical method validation

The LC-MS/MS methods with bilastine-d6 as IS were validated for all rabbit matrices (conjunctiva, cornea, aqueous humor, vitreous humor, iris/ciliary body, retina/choroid, crystalline lens, and plasma) according to the European Medicines Agency (EMA) guideline on bioanalytical method validation (EMEA/CHMP/EWP/192217/2009 Rev. 1 Corr. 2) and US Bioanalytical Method Validation [Food and Drug Administration (FDA) Guidance for Industry, May 2018].

Validation of the method included an evaluation of the following characteristics: selectivity, calibration curve performance, precision and accuracy, matrix effect, carryover, and analyte stability. The hemolysis effect, blood stability, and dilution integrity were also evaluated where appropriate.

### PK analysis

PK parameters were determined for each matrix by noncompartmental analysis using mean concentration values (right eyes, left eyes, and all eyes) for each matrix. Concentration and parameter values were rounded to two decimals, and coefficient of variation was rounded to one decimal.

PK analyses included determination of the maximum observed concentration (C_max_), the time to C_max_ (t_max_), and the area under the concentration–time curve (AUC_t_) from time 0 to the last measurable time point. PK analysis was performed using validated WinNonlin^®^ Professional software, version 6.3, integrated in the Phoenix^®^ Suite, version 1.3 (Pharsight Corporation, Mountain View, CA, USA).

Arithmetic mean concentrations were calculated only when *n* ≥ 3 and at least two numerical values were above the lower limit of quantification (LLOQ). Concentration values below the LLOQ or no peak were treated as zero.

## Results

### Method validation

Table 1 summarizes the results obtained in the validation of analytical methods for each matrix, according to the international guidelines on bioanalytical method validation. All the validation parameters were within the established acceptance limits, except for the freeze–thaw and long-term stability assessments for bilastine in aqueous humor.

**Table 1.** Validation Summary of the Liquid Chromatography–Tandem Mass Spectrometry Method for Bilastine in Rabbit Ocular Tissues and Plasma

	Plasma	Aqueous humor	Vitreous humor	Cornea	Iris/ciliary body	Conjunctiva	Crystalline lens	Retina/choroid
Selectivity	No interference	No interference	No interference	No interference	No interference	No interference	No interference	No interference
Carryover	Not observed	Not observed	Not observed	Not observed	Not observed	Yes (injection of 1 blank after ULOQ)	No	Yes (injection of at least 3 blanks after ULOQ)
Matrix factor (CV% of normalized matrix factor)	2.54%	7.52%	6.14%	6.61%	2.79%	7.20%	4.11%	5.68%
Matrix effect in 5% hemolyzed plasma validated	Yes	N/A	N/A	N/A	N/A	N/A	N/A	N/A
Dilution	N/A	10-fold	10-fold	20-fold	10-fold	10-fold	10-fold	10-fold
Bench top stability	Stable up to 2 h 5 min at RT	Stable up to 2 h 5 min at RT	Stable up to 2 h 15 min at RT	Stable up to 2 h 15 min at RT	Stable up to 2 h at RT	Stable up to 2 h 10 min at RT	Stable up to 2 h 10 min at RT	Stable up to 2 h 7 min at RT
Freeze–thaw stability	Stable after three freeze–thaw cycles from RT to −80°C	Failed	Stable after one freeze–thaw cycle from RT to −80°C	Stable after three freeze–thaw cycles from RT to −20°C	Stable after two freeze–thaw cycles from RT to −20°C	Stable after three freeze–thaw cycles from RT to −20°C	Stable after three freeze–thaw cycles from RT to −20°C	Stable after three freeze–thaw cycles from RT to −20°C
Short-term stability	Stable for 4 h 21 min at RT and after a freezing period at −80°C	N/A	N/A	N/A	N/A	N/A	N/A	N/A
Long-term stability	Stable for at least 35 days at −80°C	Failed (34 days)	Stable for 34 days at −80°C	Stable for 8 days at −20°C	Stable for 30 days at −20°C	Stable for 34 days at −20°C	Stable for 31 days at −20°C	Stable for 33 days at −20°C
Analytical run length	Validated for 121 injections	Validated for 58 points in the longest validation run (67 points at maximum^a^)	Validated for 46 points in the longest validation run (52 points at maximum^a^)	Validated for 73 points in the longest validation run (80 points at maximum^a^)	Validated for 73 points in the longest validation run (80 points at maximum^a^)	Validated for 78 points in the longest validation run (80 points at maximum^a^)	Validated for 79 points in the longest validation run (87 points at maximum^a^)	Validated for 54 points in the longest validation run (59 points at maximum^a^)
Autosampler stability	Stable up to 183 h 45 min at 4°C	Stable up to 148 h 16 min at 4°C	Stable up to 33 h 30 min at 4°C	Stable up to 177 h 57 min at 4°C	Stable up to 202 h 17 min at 4°C	Stable up to 175 h 40 min at 4°C	Stable up to 177 h 21 min at 4°C	Stable up to 66 h 21 min at 4°C
Blood stability	Stable for 1 h at RT	N/A	N/A	N/A	N/A	N/A	N/A	N/A

^a^ Maximum number of injections during sample analysis, including calibration standards, quality controls, and blanks.

CV, coefficient of variation; N/A, not applicable; RT, room temperature; ULOQ, upper limit of quantification.

Thus, stability might be compromised in aqueous humor and, consequently, the corresponding results should be considered with caution. Nonetheless, this could be a validation-related issue as incurred sample reanalysis results demonstrated the reproducibility of the bioanalytical results obtained for all matrices, including aqueous humor.

### PK analysis

The mean weights of the solid ocular tissues (cornea, conjunctiva, iris/ciliary body, retina/choroid, and crystalline lens) for both right and left eyes are shown in Table 2.

**Table 2.** Ocular Structure Weights

Matrix	Mean weight (SD)-R (g)	Mean weight (SD)-L (g)
Conjunctiva	0.051 (0.032)	0.025 (0.013)
Cornea	0.068 (0.006)	0.066 (0.006)
Iris/ciliary body	0.069 (0.046)	0.126 (0.066)
Retina/choroid	0.090 (0.031)	0.044 (0.012)
Crystalline lens	0.397 (0.028)	0.385 (0.020)

L, left eye; R, right eye; SD: standard deviation.

Measurable concentrations of bilastine were found in all matrices for all treated animals. Table 3 summarizes the PK parameters obtained for bilastine in ocular tissues and plasma. The results for plasma and aqueous humor were normalized by sample volume (mL), whereas for the rest of the samples, results were normalized by tissue weight (g). Bilastine was absorbed into the eye and reached maximum concentration levels at 6 h postadministration in the conjunctiva (C_max_: 2,545.04 ng/g; AUC_t_: 11,382.40 ng·h/g) and at 1 h postadministration in the cornea (C_max_: 609.11 ng/g; AUC_t_: 1,993.88 ng·h/g).

**Table 3.** Summary of Pharmacokinetic Parameters (Mean ± Standard Deviation) for Bilastine in Ocular Tissues and Plasma Following Single Topical Ocular Administration of Once-Daily 0.6% Bilastine Eye Drops in Each Eye of Male Dutch-Belted Rabbits

Matrix	C_max_^a^	t_max_ (h)	AUC_t_^b^
Conjunctiva	2,545.04 ± 5,412.14	6	11,382.40
Cornea	609.11 ± 334.36	1	1,993.88
Iris/ciliary body	45.09 ± 48.81	6	747.50
Aqueous humor	38.89 ± 24.72	1	95.91
Retina/choroid	22.22 ± 25.79	4	216.90
Plasma	6.10 ± 4.10	0.5	14.20
Vitreous humor	2.84 ± 3.62	4	12.17
Crystalline lens	0.85 ± 0.69	1	8.97

^a^ Nanogram per milliliter for plasma and aqueous humor; nanogram per gram for the rest of the matrices.

^b^ Nanogram hour per milliliter for plasma and aqueous humor; nanogram hour per gram for the rest of matrices.

AUC_t_, area under the concentration–time curve (from time 0 to the last measurable time point); C_max_, maximum observed concentration; t_max_, time to C_max_.

This was followed, in order of decreasing magnitude, and considering bilastine exposure levels over time (AUC), by the iris/ciliary body, retina/choroid, aqueous humor, plasma, vitreous humor, and crystalline lens (Table 3). No major differences were observed between the left and right eyes in any of the matrices.

**FIG. 1.**
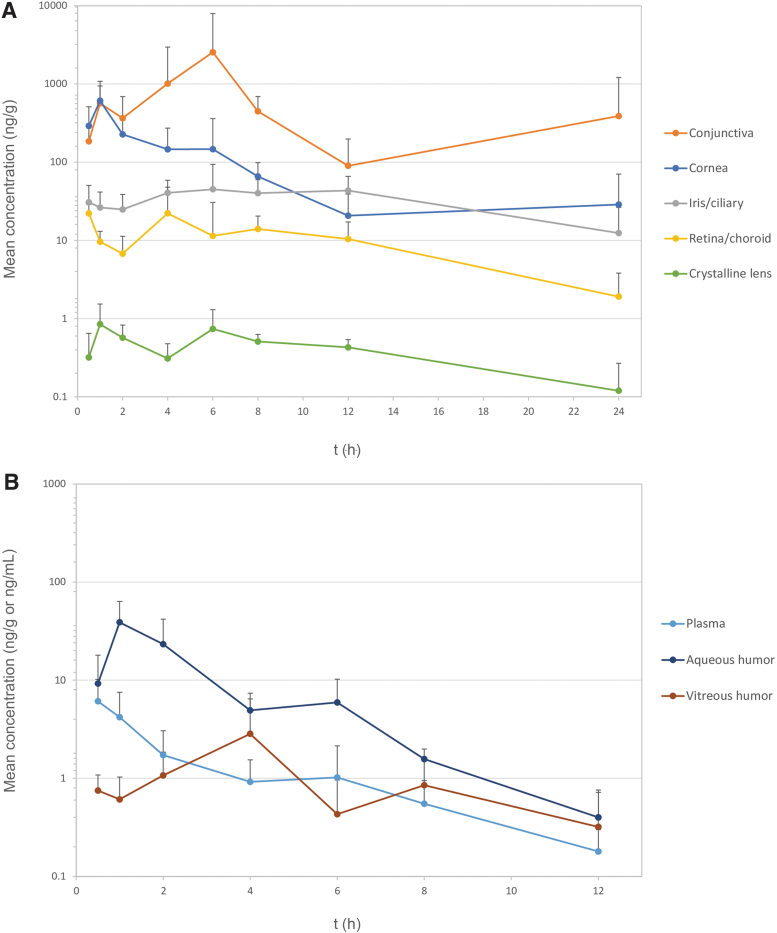
Concentration–time profiles of bilastine in log scale in the cornea, conjunctiva, iris/ciliary body, retina/choroid, and crystalline lens **(A)** and in plasma, aqueous humor, and vitreous humor **(B)** following single topical administration of once-daily 0.6% bilastine eye drops. Concentrations are in nanogram per milliliter for plasma and aqueous humor and nanogram per gram for the rest of the matrices. Bars represent standard deviations.

Mean concentrations of bilastine over time in plasma and ocular samples for both right and left eyes are shown in Fig. 1. Overall, quantifiable bilastine concentrations were observed up to 24 h postadministration in the conjunctiva (388.45 ng/g), cornea (28.68 ng/g), iris/ciliary body (12.42 ng/g), retina/choroid (1.91 ng/g), and crystalline lens (0.12 ng/g). In plasma, aqueous humor, and vitreous humor, quantifiable concentrations of bilastine were detected up to 12 h postadministration (0.18 ng/mL, 0.40 ng/mL, and 0.32 ng/g for plasma, aqueous humor, and vitreous humor, respectively).

## Discussion

In the present study, we examined the PKs and biodistribution of the newly developed, once-daily, 0.6% bilastine ophthalmic solution, following a single bilateral topical ocular administration in Dutch-Belted rabbits. The rabbit model was selected since it is considered the most appropriate animal model for evaluation of ocular kinetics given the similarity of ocular anatomy to humans, ease of handling and maintenance, and the wealth of experimental data that exist on several rabbit strains.^19,20^

LC-MS/MS was preferred for being a highly sensitive, reliable, and specific analytical technique that has been extensively used for PK and biodistribution studies in rabbits.^21–25^

Topical ocular drug delivery is considered an ideal route of administration for treatment of ocular diseases of the anterior segment of the eye. An ideal topical ocular drug delivery should be able to maintain the drug release and to remain in the immediate vicinity of the application site at the front of the eye.

For a better understanding of drug PKs after ophthalmic administration, it is essential to consider several parameters that determine ocular biodistribution, including ocular physiological and anatomical barriers, physicochemical properties of the molecule (size, charge, and hydrophobicity/hydrophilicity), and melanin–drug binding.^20,26^

When a drug is delivered topically in the eye, it faces a number of permeation barriers and clearance pathways. After topical instillation of an eye drop, a major portion of the instilled volume is lost at the precorneal level through the nasolacrimal duct or eliminated systemically through the conjunctival stroma, which contains blood and lymphatic vessels that clear the drug to the systemic blood circulation.^26,27^

**FIG. 2.**
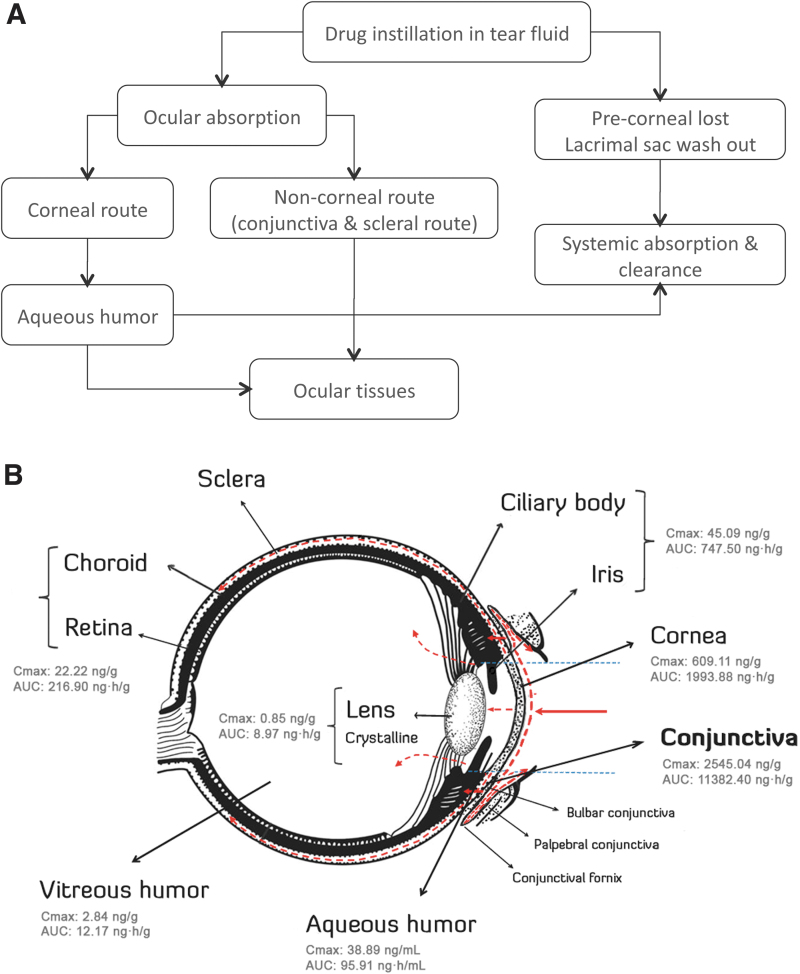
**(A)** Routes of ocular absorption. **(B)** Bilastine biodistribution in the eye structures and hypothetical route after instillation. Once bilastine reaches the ocular surface, a fraction is retained in the conjunctival and corneal tissues, whereas another fraction is cleared through the nasolacrimal drainage system or absorbed into the systemic blood circulation through the conjunctival stroma. Low bilastine plasma levels (C_max_: 6.10 ng/mL; AUC: 96.91 ng·h/mL) evidence low drug diffusion into the systemic circulation through the conjunctiva. However, the amount of bilastine that can access the bulbar conjunctiva can also enter the sclera and the ciliary body through blood vessels. The sclera is a more permeable tissue compared with the cornea, allowing easier migration of bilastine to the choroid and eventually, due to high choroidal vascularity, reaching the retina. The substantial and sustained levels of bilastine found in the iris/ciliary body and retina/choroid may partially respond to binding of bilastine to the melanin present in these pigmented tissues. Transcorneal permeation of bilastine is minimal, according to the low concentration of drug detected in the aqueous humor, vitreous humor, and crystalline lens. AUC, area under the concentration–time curve; C_max_, maximum observed concentration.

The mechanisms of ocular absorption include corneal absorption, which allows access to small ionic and lipophilic molecules, and noncorneal absorption, which permits penetration across the sclera and conjunctiva into intraocular tissues (see Fig. 2A). In addition, due to the very low permeability of the cornea, only a very small portion of the active compound will successfully reach the intraocular tissues.

Compared with the cornea, conjunctival drug absorption is generally considered to be nonproductive for intraocular drug delivery. However, it has been demonstrated that the noncorneal absorption route may significantly contribute to drug penetration into the intraocular tissue across the conjunctiva/sclera.^28^ Since the conjunctiva has a large paracellular pore density and diameter, drug absorption through this noncorneal route is preferential for compounds that penetrate the cornea poorly, such as hydrophilic molecules and macromolecules.^28–31^

After penetrating the bulbar conjunctiva, the fraction of drug that is not cleared by blood circulation can reach the scleral tissue, and following this route, access to the choroid and retina can also be granted.^32^

In our study, according to the bilastine concentration measured in each biological matrix, high (conjunctiva and cornea), intermediate (iris/ciliary body and retina/choroid), and low (plasma, aqueous humor, vitreous humor, and crystalline lens) tissue levels were categorized.

The predominant distribution of bilastine in the conjunctiva and cornea, considering both maximum concentration and exposure, corresponded well with the site of topical dosing. Nonetheless, the highest and more prolonged concentration of bilastine was observed in the intended target tissue (i.e., the conjunctiva). Interestingly, the conjunctiva/cornea AUC ratio was higher for bilastine (⁓5.7) than for other antihistamines such as olopatadine (⁓1), cetirizine (⁓0.6), and azelastine (⁓0.3).^25,33,34^

The drug level in aqueous humor is used occasionally as a surrogate marker for ocular penetration and efficacy.^35^ However, a high degree of penetration into this matrix does not appear to be essential for efficacy of antihistamine eye drops.^25,34^ The current PK work on bilastine suggests that higher drug levels in the conjunctiva than in the cornea could be associated with clinical efficacy. This differential distribution of bilastine toward the conjunctiva might have been influenced by its higher molecular weight (463.6 g/mol) and higher polar surface area (67.6 Å^2^), compared with olopatadine (337.4 g/mol; 49.8 Å^2^), azelastine (381.9 g/mol; 35.9 Å^2^), and cetirizine (388.9 g/mol; 53.0 Å^2^).

Moreover, since bilastine is a substrate for the transporter, P-glycoprotein (ABCB1 or P-gp), its distribution could have been modulated by this ATP-dependent efflux pump, especially in the cornea—where P-gp action is more efficient given its reduced permeability—and in the conjunctiva—where the expression of P-gp is significantly increased.^36–39^ This might also explain the elevated bilastine conjunctiva/plasma AUC ratio (>800) observed since P-gp efflux could limit its systemic clearance, while sustaining high levels of the drug in the conjunctiva.

In fact, unlike other antihistamines, the initial levels of bilastine in the conjunctiva did not drop rapidly, and C_max_ was reached at later time points: maximum concentrations of bilastine were observed at 6 h postadministration, whereas C_max_ levels of both 0.2% and 0.77% olopatadine ophthalmic solutions,^25^ 0.24% cetirizine,^33^ 0.1% azelastine,^34^ and 0.04 mg levocabastine eye drops^40^ were attained at 0.5–2 h. In addition, high levels of bilastine were found in the conjunctiva after 24 h of a single bilateral topical administration (388.5 ng/g, equivalent to 837.9 nM, assuming a density of 1 g/mL).

Considering that the concentration of bilastine found in the target tissue of rabbits was considerably higher than the K_i_ value for the human histamine H_1_ receptor (8.7 nM), along with the results of previous kinetic studies showing a long residence time of bilastine at this receptor,^41^ a long-lasting ocular therapeutic effect of bilastine in humans is expected.

On the other side, although bilastine required a long time to reach maximal concentrations in the conjunctiva, two clinical trials [a single-center, phase II dose-ranging study (ClinicalTrials.gov identifier: NCT03231969); and a multicenter, phase III efficacy study (ClinicalTrials.gov identifier: NCT03479307)] conducted to assess the efficacy of the bilastine ophthalmic solution for treatment of signs and symptoms of allergic conjunctivitis showed that bilastine has a rapid onset of action.

The intermediate levels of bilastine observed in the iris/ciliary body and retina/choroid support the noncorneal permeation route through the conjunctiva and reveal the potential role of melanin binding in the PKs and biodistribution of bilastine. In fact, it is well known that differences in melanin content of ocular tissues (mainly distributed in the iris, ciliary body, choroid, and retinal pigment epithelium) may alter the PKs of drugs that bind to melanin by increasing the concentration levels and prolonging the effect of the drug.^42,43^

Furthermore, an *in vitro* melanin binding assay has shown low to moderate affinity of bilastine to synthetic melanin (binding <30% at 1 and 100 μM, unpublished data). These findings could explain the substantial and sustained levels of bilastine found in the iris/ciliary body and retina/choroid.

On the other hand, bilastine levels in the aqueous humor were 15.7-fold C_max_ and 20.8-fold AUC lower than in the cornea, suggesting poor transcorneal diffusion of bilastine. These results are comparable with those obtained in rabbits for other ophthalmic antihistamines such as 0.2% and 0.77% olopatadine,^25^ 0.24% cetirizine,^33^ and 0.1% azelastine.^34^

The low levels of bilastine found in the crystalline lens and vitreous humor further support the reduced transcorneal permeation hypothesis, suggesting that the preferential absorption route of bilastine is the conjunctival pathway. A schematic representation of bilastine biodistribution in the eye and the suggested route after topical instillation is provided in Fig. 2B.

Finally, the reduced, but detectable, levels of bilastine in plasma also reinforce the idea of the bulbar–stromal conjunctival route. Despite the obvious differences between preclinical and clinical studies, low levels of bilastine in plasma are comparable with those detected after a single topical administration of 0.6% ophthalmic bilastine solution in a phase I study in healthy volunteers (C_max_: 2.79 ng/mL, AUC: 19.54 ng·h/mL; EudraCT No.: 2018-001504-11).

In addition, the comparison with previous studies of a therapeutic 20 mg oral dose (C_max_: 268.3 ng/mL and AUC 1,350.4 ng·h/mL, respectively) implies a negligible potential for systemic side effects and a large safety range in ocular administration (EudraCT No.: 2017-003787-12).

## Conclusions

The present work represents the first report of the PK and biodistribution profile in rabbits of the newly developed, once-daily, 0.6% bilastine preservative-free eye drops. The results obtained suggest that the preferential absorption route of bilastine is the conjunctival pathway, its target tissue. Since significant levels of bilastine were observed in the conjunctiva up to 24 h after dosing, this formulation can be considered suitable for once-daily administration in the treatment of allergic conjunctivitis.
